# A feasibility evaluation of Discovery Group: determining the acceptability and potential outcomes of a patient-led research group in a secure mental health inpatient setting

**DOI:** 10.1186/s40900-021-00310-0

**Published:** 2021-09-25

**Authors:** Anne Aboaja, Oluwatosin Atewogboye, Mudassar Arslan, Lucia Parry-Newton, Lindsey Wilson

**Affiliations:** 1 Forensic Service, Roseberry Park Hospital, Tees, Esk and Wear Valleys NHS Foundation Trust, Middlesbrough, UK; 2 Mental Health and Addictions Research Group, Department of Health Sciences, University of York, York, UK

**Keywords:** Patient involvement, Forensic psychiatry, PPIE, Recovery, Mental health, Secure service

## Abstract

**Background:**

Patient and public involvement and engagement (PPIE) is recognised as an essential part of health research. In addition to providing an opportunity for patients to shape health research and acquire research skills, in the inpatient mental health setting, PPIE may have additional value in providing meaningful activity and enhancing recovery, as defined using connectedness, hope, identity, meaning and empowerment (CHIME) principles. However, there have been challenges in applying PPIE principles in secure mental health inpatient settings. An eight -session PPIE programme (“Discovery Group”) was designed to support patient-led research in a secure mental health hospital. This feasibility study aims to evaluate the acceptability of the programme from the perspective of patients and identify potential outcomes.

**Methods:**

A retrospective single-arm post-programme evaluation of Discovery Group was undertaken. Participants attended an evaluation workshop where they were interviewed individually to complete an acceptability questionnaire designed using the domains of the Theoretical Framework of Acceptability. Participants also completed an outcomes questionnaire, which included CHIME-based recovery items, and were invited to share their ideas for programme improvement on posters. Quantitative data were analysed descriptively. Direct content analysis was applied to qualitative data.

**Results:**

In our sample, eight participants attended at least one session of the discovery group with one patient attending all sessions. Most of the participants felt positive about taking part in the group and expressed interest in joining another group in future. All participants experienced some burden from the effort required during group sessions, but a low level of opportunity cost in terms of the extent to which they perceived they had to forfeit benefits to participate in the programme.. Some described the group as effective in helping them learn about research. Of the five CHIME recovery domains, only connectedness was reported as a benefit of the group. The participants valued the opportunity to use their time well and demonstrate that they were undertaking purposeful activity as part of their rehabilitation and recovery.

**Conclusions:**

Discovery Group is a tool to overcome barriers to effective PPIE in research in a secure inpatient mental health setting. The programme has a high level of acceptability among participants and offered several potential outcomes which require testing through further study.

**Supplementary Information:**

The online version contains supplementary material available at 10.1186/s40900-021-00310-0.

## Plain English Summary

Patients make an important contribution to research. However, it can be hard for patients in a secure mental health hospital to be involved in doing research. So we designed a programme called Discovery Group to give patients a chance to learn about research by doing it themselves with support. Then we did this study to answer three questions:

1. Did patients find Discovery Group acceptable?
2. What are the benefits of Discovery Group?
3. How can it be improved?

Each patient completed two questionnaires about their experiences of Discovery Group. The first questionnaire asked about their level of satisfaction and how acceptable they found it. The second questionnaire asked about the benefits of Discovery Group. Patients also gave ideas for improving Discovery Group.

Overall, the patients found Discovery Group acceptable and worthwhile. They would all recommend it to other patients. However, they felt that had to put in quite a bit of effort. Patients learned about research and felt valued as fellow researchers. Discovery Group also helped them feel more confident and connected but did not help them feel more hopeful or empowered. Patients suggested using the computer during Discovery Group for a better experience.

The study showed that Discovery Group allows patients in a secure mental health hospital to be involved in research in an easy and acceptable way. There may be benefits for patients who join Discovery Group. We hope to improve Discovery Group and see if it can offer more benefits for more patients.

**Supplementary Information:**

The online version contains supplementary material available at 10.1186/s40900-021-00310-0.

## Background

Patient and public involvement and engagement (PPIE) is increasingly a focus and expectation of health research, with funding institutions such as the National Institute for Health Research (NIHR) driving this agenda forward [15]. PPIE is described as a pioneering partnership due to the “*unique and invaluable insights that patients, carers and the public are able to offer*” in the advancement of all research carried out in the health and social care sector [12]. At the lowest level of involvement, patients are consulted to inform research design but the researcher retains the dominant voice, whilst medium level involvement invites shared decision-making and an opportunity to equitably co-produce research [8]. At the highest level of PPIE, research is led by patients who have the dominant voice over non-patient researchers in some stages of the research cycle. [8]. For example, the patients may prioritise the research area for study [1] or take the lead in designing the participant information sheet.

Whilst patient involvement in health research is not a new concept in forensic psychiatry, there are ongoing challenges to achieving the levels of involvement that would result in meaningful and non-tokenistic co-production of knowledge and evidence [6, 15]. Ongoing affective or psychotic symptomatology can make it difficult for some patients to engage in activities that involve leaving one’s bedroom, listening to others, expressing one’s views and attending a fixed appointment. Barriers to facilitating PPIE in secure mental health services specifically include researcher difficulties in gaining entry to a secure building and accessing patients; failure to meet communication and literacy needs of patients [6, 17]; a large power gap between researcher and patient; lack of patient training in research skills and unresolved tensions between researchers and staff about risk management and security [17]. Despite these barriers, and beyond the direct benefits for researchers and the research itself, PPIE can provide patients with personal benefits such as: the acquisition of research knowledge and skills, opportunities to work with others, and increased confidence [6, 17]. We hypothesise that these benefits may contribute to patient recovery.

A conceptual framework of personal recovery for mental health patients has identified five recovery processes (or factors): Connectedness; Hope and optimism about the future; Identity; Meaning in life; and Empowerment, collectively known by the acronym CHIME [9]. Mental health services aim to embed these factors in all aspects of patient care and they can be used to measure change over time. It has been recommended that evaluations are undertaken to measure the impact of all working practices and therapeutic activities on these five CHIME factors [9]. It is therefore important that any PPIE activity undertaken in the mental health inpatient setting is evaluated for its evidence to support, rather than hinder, the recovery and wellbeing of involved patients.

Boredom is a commonly reported problem in inpatient mental health settings. It is a complex and subjective experience reportedly linked to mostly negative mental health recovery and outcomes such as elevated levels of hopelessness, suicidal ideation, violence and aggression [3, 10], with less frequent positive report of increased curiosity and creativity [10]. It is proposed that inpatient boredom might be overcome by the provision of a range of meaningful activities [10], even PPIE activity, in which patients are motivated to participate if they consider such activity to be meaningful [2].

Anecdotal evidence from patients on a low secure ward for adults with mental disorders highlighted their concerns about increased boredom during the first national lockdown related to the Covid-19 pandemic the lockdown. This inspired the development of “Discovery Group”, a high level PPIE programme providing experiential research skills training (for example, the research cycle, teamwork, data collection, thinking and communication) as a meaningful activity that might also have a positive impact on CHIME recovery factors and reduce boredom, whilst also overcoming the known barriers of PPIE in a secure mental health inpatient environment (Table 1). Discovery Group sessions, each lasting one hour, were held weekly on the ward between April and May 2020 (Table 2). Using a diagram, research was explained to patients as a structured approach to discovery which involved a cycle beginning with deciding what to discover, progressing to deciding how to make the discovery, and ending with sharing the discovery with others (Table 1). Patients were guided through the research cycle by the facilitators. To help patients develop research skills, facilitators employed a combination of teaching methods including: kinaesthetic (e.g., patients collected data in pairs and cross-checked results), enquiry-based (e.g., patients were encouraged to ask questions about their topics of interest to identify a research question); differentiated instruction (e.g., at the data analysis stage, patients were offered the choice of undertaking easier or more challenging tasks) and, to a lesser degree, direct instruction.

**Table 1:** Overview of the patient-led research programme

Overview of Discovery Group
All patients on the secure ward for adults with mental disorders were invited to voluntarily attend a new group in which they would learn about research and have the opportunity to carry out their own research. The group was facilitated by a clinician with research interests and co-facilitated by ward clinical staff available on the day. All staff members involved in facilitating the group were therefore known to patients. During the programme, patients were referred to as “researchers”
*Learning objectives of Discovery Group*
At the start of the programme, patients were informed of the following learning objectives:
1. To identify something new to “discover” (the research question)
2. To work out how to discover it in the current setting (the method) in a safe, ethical and confidential way (i.e., no personal information will be collected about patients or staff or anyone else)
3. To collect information to discover it (data collection)
4. To see what has been discovered (results)
5. To decide what the discovery means for us and others (interpretation and implications)
6. To agree with whom the discovery will be shared (dissemination)

**Table 2:** Content structure of Discovery Group

Programme structure		Example of session plan (Week 2)
Session	Session focus	Summarise previous sessions Thoughts or questions Review actions from the previous session Introduction to research cycle Introduction to research ethics Consider dissemination plan Identify research question Consider accessible data sources Thoughts or questions Agree actions
1	Identifying the overall research topic	
2	Introduction to research cycle/Refine research question	
3	Confirm objectives and develop methodology	
4	Data collection	
5	Data collection/analysis	
6	Data collection/analysis	
7	Data analysis/Data interpretation	
8	Data interpretation	

In July 2020, a retrospective feasibility evaluation of Discovery Group was undertaken. This evaluation aims to assess the feasibility of Discovery Group through the following objectives:

1. To measure and understand attendance at Discovery Group.
2. To determine the acceptability of Discovery Group to patients.
3. To identify potential outcome measures.
4. To identify areas for further development of Discovery Group.

## Method

### Design

A retrospective single-arm post-programme evaluation was undertaken of this pilot programme. An additional file describes the level of patient involvement in this study [see Additional file 1].

### Participants and recruitment

Patients who had not attended any Discovery Group sessions were invited in person to voluntarily contribute to the evaluation individually outside of the workshop. All patients who had participated in at least one Discovery Group session were invited to participate in the evaluation workshop for participants. A member of the evaluation team met individually with each patient to explain the purpose and structure of the evaluation workshop and the voluntary nature of participation.

### Instruments

To measure and understand recruitment to Discovery Group from the perspective of non-participants, individual interviews were conducted for patients who had not attended Discovery Group sessions using a brief questionnaire comprising four questions: (1) Have you heard of Discovery Group? (2) Can you tell me what Discovery Group is about? (3) Can you tell me why you were not part of Discovery Group? (4) Would you be interested in joining Discovery Group in the future? Prompts were given to elicit more detailed responses.

The following instruments were used to obtain information from participants during the workshop:

I. *Acceptability Questionnaire (to determine the acceptability of Discovery Group to patients)*. The principles of the Theoretical Framework (TFA) guided the development of a question to determine the acceptability of Discovery Group to participants. The TFA is a construct of multiple domains: affective attitude, burden, ethicality, intervention coherence, opportunity costs effectiveness, effectiveness and self-efficacy. Collectively these seven domains describe the extent to which recipients of an intervention cognitively and emotionally experience it to be acceptable [14]. The TFA does not exist in the form of a validated instrument with set questions. Therefore, relevant questions were derived from each domain of the framework. The Acceptability Questionnaire also included questions about attendance which further helped to measure and understand recruitment to the programme from the perspective of participants. The Acceptability Questionnaire was completed with participants by workshop facilitators during individual interviews.
II. *Outcomes Questionnaire (to identify potential outcomes)*. This questionnaire was developed to identify a range of potential positive outcomes which participants experienced and attributed to their participation in Discovery Group. Outcomes included intended benefits identified during the development phase of the programme. Participants were asked to rate on a four-point Likert scale the extent to which they perceived Discovery Group had helped them to achieve a desired outcome. Response options ranged from “did not help at all” to “helped a lot”. Participants completed this individually, with assistance from a workshop facilitator as required.
III. *Programme improvement posters (to identify areas for further development of Discovery Group)*. Three posters displayed on a wall were used to generate specific feedback on Discovery Group, including areas for development. Participants were invited to independently write their individual comments and responses to each of the following three questions appearing on the posters: (1) How could we make Discovery Group better? (2) What did you like most about Discovery Group? (3) What were the main things you did not like?

### Workshop structure

The workshop was held in the communal dining area of the ward. Drinks and pre-served plates of hot and cold buffet food were provided. Participants moved between the various stations to complete the questionnaires and provided feedback on the posters. After the workshop, all Discovery Group participants were thanked, congratulated and formally presented with certificates of attendance during a ward community meeting.

### Analyses

Data pertaining to the acceptability of Discovery Group were analysed using direct content analysis [7], using TFA domains as pre-determined codes. Where appropriate, frequency counts of this qualitative data were conducted. Descriptive statistics were undertaken of quantitative data describing potential outcomes. Themes for developing the programme were identified through conventional content analysis of poster responses [7].

## Results

### Overview of research undertaken by patients attending Discovery Group

Patients chose to study the frequency and profile of Covid-19-related reporting of different countries in UK newspapers. Three patients attended the first session in which the overall research topic was chosen. Research findings were prepared for public dissemination.

### Attendance at Discovery Group

There were 18 patients on the ward, all of whom were invited to join Discovery Group. Of the ten patients on the ward who chose not to participate in Discovery Group, nine agreed to be interviewed as part of the evaluation. Of those interviewed, six reported awareness of Discovery Group. Six cited lack of interest as the reason for not attending the group. One patient could not recall his reason for not attending. Two patients, who stated they had not attended due to lack of awareness of the group or believing they were ineligible to attend, reported they would be interested in joining the group in the future, whilst the remaining patients did not intend to do so.

Of the 18 patients invited, eight patients attended at least one of the Discovery Group sessions. One person attended all eight sessions, while the remaining participants missed at least one session. Reasons given for missing sessions were joining the programme after the first session and having other appointments at the same time. All eight patients chose to participate in the evaluation study.

### Acceptability of the Discovery Group intervention to participants based on the seven domains of the theoretical framework of acceptability

#### Domain 1: affective attitude

This domain describes how a participant felt about the programme. Six (75%) participants felt positive about the group after taking part, whist the remainder felt neither positive nor negative. Enjoyment of an aspect of the intervention explained the positive feelings of the six participants. Participants with a positive experienced affective attitude to Discovery Group mostly enjoyed the learning (mentioned four times) and the elements of the group context (mentioned three times). One person also mentioned the novelty of the intervention as a factor influencing him to feel positive towards the programme. Although two (25%) participants stated they would not join another Discovery Group in the future (“Did not enjoy” and “Think I’ve took my turn, other people should do it for motivation”), all participants reported they would recommend the programme to another patient.

“It wasn’t just me doing it, liked working in the group and mentally challenging learning new things about Covid-19.”

“Was good information. Enjoyed group as was something different.”

“I learnt something new.”

All except one participant named a favourite aspect of Discovery Group. There was evidence that learning and undertaking research (including hypothesis testing) were enjoyable to most participants. The use of “we” and “our” in the context of participants obtaining a benefit from a shared experience indicates that there may have been a therapeutic effect of the group.

“I enjoy learning new information”

“When we came to conclusion of the research and finding out results – felt as if we had achieved something…”

“Seeing how our work became a graph, i.e., results – could see our work come together and hadn’t wasted time.”

“Finding out which country had highest infection rate, to see if what I thought would be right.”

In contrast, the least favourite aspects of Discovery Group were more varied and included dislike of the teaching style, dissatisfaction with the venue, and distress caused by data collected.

“Being questioned at conclusion what I’d just been reading, felt like it was an exam.”

“The amount of deaths I find out about, saw people who were dying, upset me.”

“Writing – don’t like writing as find it difficult.”

“Noisy in dining room, other patients who weren’t participating weren’t quiet and watching TV.”

#### Domain 2: Burden

This domain describes the amount of effort the participant perceived was necessary to be part of the programme. Attending Discovery Group placed up to moderate, but not excessive, levels of experienced burden on all participants who reported effort was required for reading, listening, writing, collecting data and interpreting results. Two participants reported that effort was required to concentrate during sessions.

#### Domain 3: Ethicality

The ethicality is the extent to which programme had a good fit with participant’s value system. Participants valued not only the opportunity to use their time well and develop relationally, but also to demonstrate to staff that they were undertaking purposeful activities as part of rehabilitation.

“Make me look motivated for doctor.”

“Seeing [name of lead facilitator], enabling me to talk to her more.’

“Building relationships.”

“Felt I have more confidence in group settings.”

“Recognition for joining in.”

“Tick boxes.”

#### Domain 4: Intervention coherence

This domain reflects the extent to which the participating individual understood the programme and how it worked. High intervention coherence was evident in responses indicating that most participants had a good understanding of Discovery Group, with seven participants describing the intervention using phrases related to the aspects of the research cycle whilst one participant referred only to the to the research topic, Covid-19. Those who understood the intervention were also aware of how Discovery Group helps people understand the research cycle:

“Learned by doing.”

“It takes you through the steps so you know what research is about.”

“Broke research cycle down so could explain.”

Whilst half of the participants believed Discovery Group had been developed and implemented to provide a learning opportunity, the remaining participants thought the motivation was to provide them with a purposeful activity:

“[Lead facilitator’s name] wanted us doing something instead of sat doing nothing.”

“To occupy our time, give us something to do.”

Overall, among participants there was an understanding of the expressed objectives of the group (learning) as well as awareness of the motivation for its development (to address potential boredom).

#### Domain 5: Opportunity costs

The opportunity costs are the extent to which the participant perceived he had to give up benefits in order to take part in the programme. All participants experienced the opportunity costs of Discovery Group to be low. None felt they had to give up activities to attend. If not attending Discovery Group, most participants reported they would have spent the hour engaged in entertainment activities, especially watching television, which was mentioned by five participants. Once participants joined Discovery Group, even if they did not enjoy the group, they reported having missed sessions only because of conflicting appointments such as reviews with clinical staff or due to poor physical health.

#### Domain 6: Effectiveness

This domain describes the extent to which the participant felt the intervention was effective. Largely, participants perceived the group to have achieved its intended purpose. They most commonly reported that Discovery Group was most effective in providing a stimulating opportunity to learn about and do research, and an activity to occupy time. All participants reported they had felt like a valued researcher in the group whose opinion really counted.

“More familiar with research.”

“I know how to break things down.”

“Spent time productively.”

“Filled the time in.”

“Provided a bit of stimulation.”

#### Domain 7: Self-efficacy

This domain describes the confidence the participant had that he could perform whatever was required to take part in the group. Most participants felt either very confident (n = 3) or somewhat confident (n = 4) to some degree that they could undertake the tasks required to participate in the group; one participant lacked self-efficacy in this area. One participant rated himself as having performed very well during sessions; three thought they had performed badly.

### Potential outcomes of Discovery Group

**Fig. 1:**
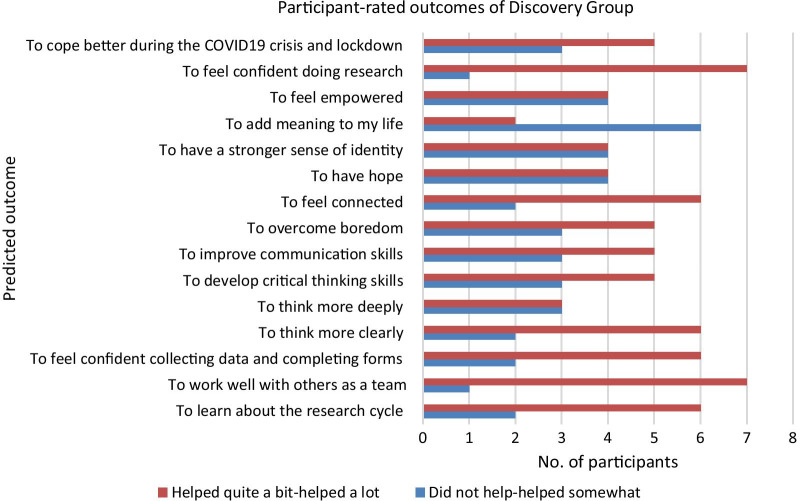
Participant-rated outcomes of Discovery Group

Figure 1 shows that several potential beneficial outcomes were confirmed by participants who had attended the group. Seven out of eight participants reported that the group helped them to feel confident doing research and to work well with in a team with others. Six (75%) of participants reported that Discovery Group helped them to learn about the research cycle and feel confident collecting data and completing forms, and to think more clearly. Five participants reported that the group helped them to overcome boredom, cope better during the Covid-19 crisis and lockdown, improve their communication skills and develop critical thinking skills.

Six participants reported that the group did not help to add meaning to their lives whilst an equal number reported that it helped them feel connected. Four participants reported that the group was helpful in increasing the remaining three CHIME principles (hope, identity and empowerment) whilst an equal number found that the group was not helpful in addressing these domains.

Of the seven predicted potential outcomes of the programme that would benefit participants, more than half of the participants confirmed six outcomes: teamwork (n = 7), learning about the research cycle (n = 6), data collection and completing forms (n = 6), recovery—connectedness (n = 6), critical thinking (n = 5), written/spoken presentation skills (n = 5) and overcoming boredom (n = 5). There was less evidence from participants to support additional potential benefits in the recovery processes of hope, identity, meaning and empowerment.

### Areas identified for development of Discovery Group

When asked, “What could we do to make Discovery Group better?”, one participant stated that no changes were necessary. The remaining seven participants offered a range of suggestions relating to: structure, environment, resources, level of involvement and research theme. Table 3 illustrates the main areas for improvement.

**Table 3:** Participant-reported areas for development of Discovery Group

Area for further development of Discovery Group	Illustrative quotes
Increase group size	“More people to take part”
Select environment more conducive to learning	“Run it in a quiet room or office like room” “Presentation—better environment perhaps?”
Vary resources used	“Use of computer equipment”
Increase learning opportunities	“Spread the workload out more so a wider range of research can be done” “Some interesting homework to do through the week (perhaps optional)” “Possibly create the graphs ourselves”

## Discussion

### Main findings in relation to evaluation aim/objectives

A novel eight-session programme, “Discovery Group”, designed to offer a high level of PPIE in research as an activity that might provide research skills, improve CHIME recovery factors, and relieve boredom was piloted among a group of patients on a secure mental health ward. Subsequently, a workshop was held with patients to evaluate the feasibility of the programme. Although Discovery Group did not attract the attendance of all patients on the ward who were aware of the programme, it was found to have a high level of acceptability overall to patients who participated, reflected by the finding that all participants stating they would recommend the group to other patients. Participants were mostly positive about programme, found it coherent and effective, and felt they could attend at low opportunity cost. Discovery Group offered a number of potential outcomes of benefit for participants, particularly in acquiring confidence and skills in research and teamwork and feeling connected. However, most CHIME recovery factors such as empowerment and meaning, were not found to be potential benefits. Most participants felt positive about the group but also identified areas for programme development.

### Contextualisation of main findings in relation to the literature

Consistent with a previous study of patients’ interest in being involved in research, participants were motivated to participate in the Discovery Group research group for a range of individual reasons [4]. Previous studies have also reported that the acquisition of knowledge is a strong motivator for patients [2, 4]. It is highly notable that participants chose to focus their research in the fields of infectious diseases and journalism, namely Covid-19 and newspapers. Qualitative research has shown that patients are often interested in researching their own diseases [4], yet in Discovery Group participants did not wish to investigate psychiatric conditions. It is possible that, rather than having a research interest in their own diagnoses, patients are interested in topics that are most relevant and important to them. Whilst this might otherwise have been the illness linked to current treatment, it appears that among participants in the present study, the Covid-19 pandemic was of greatest important to participants who attended Discovery Group during the Covid-19-related lockdown that prevented routine visits from their families to the hospital and imposed significant restrictions on their daily routines.

Outside of a global pandemic, the patients might still have formulated a research question unrelated to psychiatry. It has been acknowledged that patients have research interests that are not limited to their mental illness [5]. Discovery Group provides users of mental health services with the unique opportunity to be involved in research simply as representatives from the lay community rather than being linked to an illness. An alternative explanation for the participants’ preference to undertake non-psychiatric research is that within secure inpatient settings therapeutic interventions such as psychoeducation provide patients with an alternative opportunity to discover more about their own diagnoses.

Recognised barriers to PPIE within mental health include patients’ experience of not feeling they are equal partners to researchers [11]. However, Discovery Group was able to overcome this potential barrier by creating a positive environment in which all participants felt valued as researchers and believed their opinions counted. In this way, the risk of tokenistic research involvement was minimised. The involvement of facilitators who worked within the secure hospital and were known to patients attending the group overcame potential barriers that have been faced by external non-clinical researchers who may encounter delays in accessing secure areas and establishing relationships with staff and patients [6, 17]. However, that the facilitators were members of the ward clinical team may have influenced the decision for patients to attend, especially if they were fearful of displeasing their treating team, thus introducing the potential risk of unintentional coercion. In addition to the alleviation of boredom and a greater sense of connectedness, patients reported the benefit of acquiring knowledge of the research cycle and developing research skills, the lack of which has been reported as a barrier to PPIE in secure settings [17]. The findings do not indicate that this PPIE programme might adversely affect patient recovery. Lastly, the experience of participating in Discovery Group may inspire patients to be involved in future research [16].

### Strengths and limitations of the evaluation

To our knowledge, this is the first patient-led research programme designed for use in secure inpatient mental health settings. The high response rate (100%) to the invitation for patients who had attended the Discovery Group to participate in the evaluation workshop is a particular strength of the study. However, there are a number of limitations to this pilot feasibility study. The sample size was small. Whilst this was primarily due to the overall size of the ward and the low proportion of patients who chose to attend Discovery Group, Covid-19-related restrictions precluded the use of a larger space that could safely accommodate more than nine patients. The study was designed to evaluate the acceptability of Discovery Group to patients and understand motivations to attend. This was achieved by interviewing not only patients who chose to attend Discovery Group, but also those who did not attend at all. Although not within the scope of the aims of this evaluation, programme acceptability to staff who facilitated the group and provided support to patients during sessions is also important and should be considered in a follow-up study [14]. Although the design of the Acceptability Questionnaire was grounded in the Theoretical Framework of Acceptability [14] and the Outcomes Questionnaire based on the potential outcomes determined during the developmental stage of Discovery Group, neither instrument had been validated. However, there is potential to further develop and validate the Acceptability Questionnaire to evaluate a range of interventions for patient involvement in research and service improvement in healthcare settings. In the absence of pre- and post-testing and comparison with a control group, it is not possible to ascertain whether the reported programme outcomes identified retrospectively by participants were attributable to Discovery Group or other influences not measured in the present study. Furthermore, the study design did not allow exploration of the individual components of the programme which might be linked to the potential outcomes. However, triangulation of the prospectively stated intended outcomes with the retrospective quantitative and qualitative data from participants strongly suggests which outcomes should be studied in a future evaluation. Finally, although PPIE was a motivation for conducting this research, there was a notable absence of patient involvement in designing, implementing and reporting this evaluation.

### Ethics of the patient-led research group

An interesting ethical question the authors faced was whether the patient researchers should receive payment for their involvement in research. Best practice guidance recommends that patients who are involved in research receive appropriate payment in recognition of their time and contribution [13]. Whilst the patient researchers spent up to eight hours undertaking research during sessions, Discovery Group was established primarily as a therapeutic, educational and recovery-focused intervention, in which both attendance and participation were voluntary. It was acceptable for a patient to attend and observe the research process without making a contribution. Ownership was heavily weighted towards the patients; if the patient researchers chose not to undertake the research, the study would not have been completed. In the inpatient setting, patients are not remunerated for attending therapeutic and educational groups. The authors argued that it was therefore neither necessary nor appropriate to offer payment to patients who attended Discovery Group. Furthermore, the involvement of the patient researchers was appropriately recognised through the public presentation of certificates and the invitation to co-author or be acknowledged in publications arising from the patient-led research [16]. This approach addressed the negative experience of lack of acknowledgement previously reported by patients involved in research [2].

### Future development of Discovery Group

Based on participant responses, the group should be held in a quieter environment to avoid distractions. Whilst patients might prefer more participants in the group, the maximum number who could be safely accommodated is likely to be restricted by social distancing requirements. In response to patient feedback on the high level of concentration, the session plan should be developed to include breaks and, where possible, patients should be offered the choice of higher or lower intensity research tasks within sessions. Future participants are likely to value the opportunity to be involved in creating graphs. This could be achieved by offering a computer-based tutorial on creating tables and graphs for interested patient researchers. Providing participants with a greater degree of responsibility and ownership of the research might increase the level of the CHIME factor empowerment experienced through Discovery Group. Given the feedback that one patient found the subject upsetting, in future a safety plan should be incorporated into the design of the programme to ensure adequate support is available. The form of support may depend on the research topic selected by patients but might include offering time for debriefing at the end of each session. At the end of each session, the option of undertaking a between-session task should be offered for those keen to extend the learning period. This would also be an opportunity for patients to continue working on tables and graphs under supervision outside of the sessions. Further patient involvement opportunities should be offered during the next phase of programme development.

## Conclusions

Discovery Group is a novel intervention that offers high-level, non-tokenistic PPIE suitable for use in secure mental health inpatient settings. It produces research of value to patients through a programme of high acceptability and provides them with potential benefits of recovery as well as research knowledge and skills, and an activity that alleviates boredom. In addition, participants enjoy leading the research at different stages and feel valued as researchers with important opinions. The design of the programme overcomes a number of barriers previously reported by researchers attempting PPIE in a secure mental health hospital environment. Furthermore it shows promise of a model that enhances autonomy, and breaks down some important power and paternalistic barriers that can be experienced by patients detained in secure mental health settings [17]. Finally, a future evaluation study that involves patients during the design, implementation, evaluation and writing stages, aiming to measure the potential outcomes identified in the present study using pre- and post-testing with a control group would reliably demonstrate the effectiveness of the revised Discovery Group and ensure meaningful involvement with patients as co-researchers.

## Supplementary Information

**Additional file 1**: GRIPP2 short form for Discovery Group Evaluation. Description of data: This file contains a detailed description of patient involvement in the various stages of the research process for the present evaluation study. It includes critical PPI reflections arising from the study.

## Data Availability

The datasets generated and/or analysed during the current study are not publicly available due to the necessity not to compromise the individual privacy of participants. Furthermore, participants did not give consent for data provided to be made publicly available in this way.

## References

[1] Aboaja A, Forsyth B, Bates H, Wood R (2020). Involving service users to identify research priorities in a UK forensic mental health service. BJPsych Bull.

[2] Ashcroft J, Wykes T, Taylor J, Crowther A, Szmukler G (2016). Impact on the individual: what do patients and carers gain, lose and expect from being involved in research?. J Ment Health.

[3] Bowser A, Link W, Dickson M, Collier L, Donovan-Hall MK (2018). A qualitative study exploring the causes of boredom for men with a psychosis in a forensic setting. Occup Ther Ment Health.

[4] Bradley M, Braverman J, Harrington M, Wicks P (2016). Patients’ motivations and interest in research: characteristics of volunteers for patient-led projects on PatientsLikeMe. Res Involv Engagem.

[5] Carr S (2019). ‘I am not your nutter’: a personal reflection on commodification and comradeship in service user and survivor research. Disabil Soc.

[6] Faulkner A, Morris B (2002). User involvement in forensic mental health research and development.

[7] Hsieh H-F, Shannon SE (2005). Three approaches to qualitative content analysis. Qual Health Res.

[8] Jennings H, Slade M, Bates P, Munday E, Toney R (2018). Best practice framework for Patient and Public Involvement (PPI) in collaborative data analysis of qualitative mental health research: methodology development and refinement. BMC Psychiatry.

[9] Leamy M, Bird V, Boutillier CL, Williams J, Slade M (2011). Conceptual framework for personal recovery in mental health: systematic review and narrative synthesis. Br J Psychiatry.

[10] Marshall CA, McIntosh E, Sohrabi A, Amir A (2019). Boredom in inpatient mental healthcare settings: a scoping review. Br J Occup Ther.

[11] Mellifont D (2019). Shifting neurotypical prevalence in knowledge production about the mentally diverse: a qualitative study exploring factors potentially influencing a greater presence of lived experience-led research. Can J Disabil Stud.

[12] NIHR. How we involve patients, carers and the public. Retrieved 13 Jan 2020, from https://www.nihr.ac.uk/about-us/our-contribution-to-research/how-we-involve-patients-carers-and-the-public.htm. (2020).

[13] Pollard K, Donskoy A-L, Moule P, Donald C, Lima M, Rice C (2015). Developing and evaluating guidelines for patient and public involvement (PPI) in research. Int J Health Care Qual Assur.

[14] Sekhon M, Cartwright M, Francis JJ (2017). Acceptability of healthcare interventions: an overview of reviews and development of a theoretical framework. BMC Health Serv Res.

[15] Staniszewska S, Denegri S, Matthews R, Minogue V (2018). Reviewing progress in public involvement in NIHR research: developing and implementing a new vision for the future. BMJ Open.

[16] TEWV. “Dipping our feet into research": the experiences of the Discovery Group as we analysed media coverage of Covid-19. Case study: The Discovery Group. https://rds-nenc.nihr.ac.uk/case-study-the-discovery-group/. (2021).

[17] Völlm B, Foster S, Bates P, Huband N (2017). How best to engage users of forensic services in research: literature review and recommendations. Int J Forensic Ment Health.

