# Supplementary material for: A feasibility evaluation of Discovery Group: determining the acceptability and potential outcomes of a patient-led research group in a secure mental health inpatient setting
Source: Res Involv Engagem. 2021 Sep 25;7:67. doi: 10.1186/s40900-021-00310-0 (PMC8465701; doi:10.1186/s40900-021-00310-0)
Supplement: Supplementary file 1 — Additional file 1: GRIPP2 short form for Discovery Group Evaluation. Description of data: This file contains a detailed description of patient involvement in the various stages of the research process for the present evaluation study. It includes critical PPI reflections arising from the study. [file 40900_2021_310_MOESM1_ESM.pdf]

Additional file 1: GRIPP2 short form for Discovery Group Evaluation

Section and topic	Item	Reported on page no.
**1: Aim**	This evaluation aimed to assess the feasibility of Discovery Group, an eight-session PPIE programme. Patients were not involved in setting the research question.	3,4,7,9
**2: Methods**	Patients were participants rather than research partners in this evaluation study. As participants, they contributed to the evaluation of Discovery Group and offered suggestions for improving the programme. However, they were not involved in the design of the study or in data collection or analysis.	4,9
**3: Study results**	Patients were not involved in analysing data. However, the results showed that the Discovery Group offered a high level of patient involvement.	6,9
**4: Discussion and conclusions**	This evaluation study was limited in that it did not have a high level of patient involvement, although the area of study was related to patient involvement. The findings of the study were compared with findings reported in a related blog published by patients who had participated in Discovery Group as research partners.	8,9
**5: Reflections/critical perspective**	Understanding and advancing PPI was a motivation for the study. Where a smaller scale pilot evaluation does not have a high level of patient involvement, patients who participated in the studied programme (in this case the first Discovery Programme) could be invited to be research partners in the design of future evaluation studies. In this case, patient participants could be involved in all stages of the next evaluation from refining the programme based on recommendation, setting the research question, designing questionnaires, analysing anonymised data and drafting the manuscript.	3,10
